# Endobronchial valve therapy for patients with advanced emphysema. *A report from a tertiary care center in China*

**DOI:** 10.15537/smj.2022.43.12.20220527

**Published:** 2022-12

**Authors:** Hang Yu, Zhen Yang, Minghui Zhu, Zhixin Liang, Wei Zhao, Qiang Zhu, Liang-an Chen

**Affiliations:** *From the Senior Department of Respiratory and Critical Care Medicine, the Eighth Medical Center, Chinese PLA General Hospital, Beijing, China.*

**Keywords:** endobronchial valve, emphysema, bronchoscopic lung volume reduction

## Abstract

**Objectives:**

To assess the efficiency and safety of endobronchial valve (EBV) treatment in Chinese patients.

**Methods:**

A retrospective analysis was performed in patients with chronic obstructive pulmonary disease who underwent EBV implantation in our hospital between October 2010 and January 2017. All patients were confirmed with no collateral ventilation (CV-) or with low airflow (LF) in the treated lobe. Pulmonary function parameters, the 6-minute walk distance (6MWD), the modified Medical Research Council (mMRC), as well as adverse events in the follow-up period were recorded.

**Results:**

Thirty-eight advanced emphysema patients received EBV implantation. Significant improvements were found in forced expiratory volume in 1 second (FEV_1_)(FEV_1_: +0.12 L), 6MWD (+64.9 m), and mMRC (-0.5 points). A total of 55.3% and 65.8% of subjects met the score for the minimal clinically important difference in FEV_1_ and 6MWD, respectively. FEV_1_ improved more significantly in the CV- group than in the LF group. Pneumothorax or death did not occur during the follow-up period.

**Conclusion:**

Endobronchial valve treatment in patients with advanced emphysema and CV- provides clinically meaningful benefits with a low incidence of pneumothorax. The efficiency and safety of EBV therapy are acceptable in China.

**C**hronic obstructive pulmonary disease (COPD) is defined as a severe, long-standing respiratory disease characterized by persistent limitation of airflow and worsening disease course.^1^ Chronic obstructive pulmonary disease causes persistent and progressive respiratory symptoms and therefore leads to breathing difficulties. Medication alone has shown inadequate efficacy in patients with severe COPD and advanced emphysema.^2^ Since 2003, endobronchial valves (EBV) has emerged as a potential therapeutic alternative for advanced emphysema. Recent randomized controlled trials on EBV (TRANSFORM 2017, LIBERATE 2018) had shown that patients without collateral ventilation (CV-) of the targeted lobe could benefit significantly.^3,4^ However, the proportion of patients who achieved or exceeded the minimal clinically important difference (MCID) in FEV_1_ was still unsatisfactory (only 49.9%-59.3%). Therefore, further exploration of a better patient selection criterion for EBV therapy is required.

In China, EBV was first used for patients with advanced emphysema in 2010 in our hospital. In this study, we aimed to present our experience of EBV therapy in terms of its safety and efficacy and to investigate the pre-procedure predictors in Chinese patients.

## Methods

This retrospective analysis was carried out among patients with advanced emphysema treated with EBV (Pulmonx, Inc., Redwood City, CA, USA) in our hospital in China between October 2010 and January 2017. Patients eligible for this study were those diagnosed with severe COPD and advanced emphysema. A Chartis assessment (Pulmonx, Inc., Redwood City, CA, USA) was performed on all patients in the same bronchoscopic operation before EBV treatment, and targeted lobes were confirmed to have no CV or low airflow. The other inclusion and exclusion criteria were as follows: i) aged between 40 to 75; ii) heterogeneous emphysema; iii) FEV_1_ of less than 45% of the predicted value, total lung capacity (TLC) of >100% of the predicted value, and residual volume (RV) of >150 percent of the predicted value; iv) body mass index of <31.1 kg/m^2^ (male) or 32.3 kg/m^2^ (female); v) PaCO_2_ of <75 mmHg and PaO_2_ of more than 45 mmHg (while breathing ambient air); vi) 6MWD of at least 100 m. In addition, acute exacerbation of COPD and other severe pulmonary diseases, thoracic surgery, sputum production >4 tablespoons per day, pulmonary hypertension (≥40 mmHg), fever or any other sign of an active infection, psychosis or any diseases that might fail the assessments were excluded.

This study was according to principles of Helsinki Declaration. The Ethics Committee of the Chinese PLA General Hospital approved the study (No. S2021-369-01), and the registration number was ChiCTR2100050326 in the Chinese Clinical Trial Registry. A written informed consent was obtain from all subjects to participate in this study.

Computed tomography (CT) scans (SOMATOM Definition; Siemens Healthcare, Forchheim, Germany) were performed at the breathing level of TLC at baseline and post-procedure. Quantitative CT analyses were performed at baseline and follow-up scans, using a Food and Drug Administration-approved imaging software (FACT^®^ quantitative software, De Xin, Shaanxi, China). Two radiologists independently handled the semi-automatic analyses of all scans. The fissure integrity (FI) of each lobe was also calculated automatically and assessed by an experienced radiologist. The integrity of the interlobar fissure was defined as a calculation of more than 90% of the fissure areas by the FACT^®^ software.

Clinical and radiological outcomes were evaluated. The assessment of clinical outcomes was performed by the pulmonary function test, the 6MWD, and the modified Medical Research Council (mMRC) dyspnea scale. The improvements in FEV_1_, forced vital capacity, TLC, RV, 6MWD, and the mMRC dyspnea scale were analyzed from baseline to post-procedure. A clinical response was defined when FEV_1_ or 6MWD achieved the MCID from the baseline (FEV_1_, a 15% increase; 6MWD, a 25 m increase), and the clinical response rate was calculated. All adverse events were noted during the study.

### Statistical analysis

The SPSS for Windows, version 26.0 (IBM Corp., Armonk, N.Y., USA) was used for data analysis. Continuous variables were shown as mean ± SD or median (interquartile range) whenever appropriate. The outcome variables at baseline and post-procedure were compared by Wilcoxon signed-rank test or Paired t-test. The number of patients who had achieved the MCID score for FEV_1_ or 6MWD was used to calculate response rates. The results were considered statistically significant when *p*-value of <0.05.

## Results

From 2010 to 2017, 38 patients with advanced emphysema received EBV implantation in our hospital. Before EBV therapy, optimal medical management (including COPD medication, continuous oxygen, and pulmonary rehabilitation for at least 3 months) was prescribed. The average post-bronchodilator FEV_1_, 6MWD, and mMRC scores at baseline were 0.85 ± 0.28 L, 287.06 ± 98.54 m, and 3.0 points. The interval between baseline assessments and the treatment was 11 days [6, 14 days]. Other baseline demographics and characteristics are shown in Table 1.

**Table 1:** - Demographics and clinical characteristics of subjects in baseline assessment.

Characteristics	mean±SD
Gender (male)	38 (100%)
Age (years)	62.2 ± 6.9
BMI (kg/m^2^)	20.7 [19.2, 23.9]
Smoking history (pack years)	40.0 [30.0, 60.0]
Duration of smoking abstinence (years)	4.0 [0.5, 6.0]
COPD history (years)	8.0 [6.0, 10.5]
GOLD stage	Stage III: 18 (47.4%) Stage IV: 20 (52.6%)
PaO_2_ (mmHg)	72.28 ± 8.06
PaCO_2_ (mmHg)	49.00 ± 8.50
SaO_2_ (%)	93.23 ± 2.26
Potential of hydrogen	7.37 ± 0.04
HCO_3_- (mmcl/L)	27.29 ± 4.05
Post-BD FEV1 (L)	0.85 ± 0.28
Post-BD FEV1 (% of predicted)	30.78 ± 10.22
Post-BD FVC (L)	2.27 ± 0.76
Post-BD FVC (% of predicted)	63.53 ± 20.28
Post-BD FEV1/FVC (%)	38.17 ± 9.30
Post-BD RV (L)	6.12 ± 1.43
Post-BD RV (% of predicted)	263.78 ± 62.75
Post-BD TLC (L)	8.75 ± 1.54
Post-BD TLC (% of predicted)	138.99 ± 23.81
Post-BD RV/TLC (%)	69.76 ± 9.21
6MWD (m)	287.06 ± 98.54
mMRC (points)	3.0 [2.0, 3.0]

Values are presented as mean ± standard deviation (SD) or median (interquartile range). COPD: chronic obstructive pulmonary disease, GOLD: Global Initiative for Chronic Obstructive Lung Disease, BMI: body mass index, post-BD: post-bronchodilator, FEV1: forced expiratory volume in 1 second, FVC: forced vital capacity, RV: residual volume, TLC: total lung capacity, 6MWD: 6-minute walk distance, mMRC: modified Medical Research Council, HCO_3_: bicarbonate

Under general anesthesia (28.9%) or conscious sedation (71.1%), a median of 3 valves (range, 1-6) were inserted into the bronchi of the targeted lobe for each subject. Five patients received EBV treatment in the right middle lobe alone. In addition, 71.1% of the treated lobes had no CV and 28.9% had a low flow (LF), according to the Chartis System results. Finally, 65.8% of the treated lobes had >90% FI and 34.2% of the treated lobes had <90% FI (80% - 90% FI: 5 patients; <80% FI: 8 patients).

### Clinical outcomes

The interval from treatment to follow-up assessment was 86 days [78, 97 days]. At 3 months post-procedure, a significant improvement was noted in FEV_1_ with an absolute change of 0.12 ± 0.20 L (*p*<0.001). Meanwhile, forced vital capacity, RV, RV/TLC ratio and 6MWD all showed statistically and clinically significant improvements post-procedure. (*p*<0.001). The mMRC dyspnea scale score decreased significantly (*p*=0.007) and the target lobe volume reduction was 566 ± 741 ml (*p*<0.001). A total of 55.3% and 65.8% of the subjects met or exceeded the score for the MCID in FEV_1_ and 6MWD (Table 2). Besides, the no collateral ventilation (CV-) group showed greater improvements in FEV_1_, RV, TLC, and RV/TLC. Compared with the LF group, more subjects met or exceeded the score for the MCID in FEV_1_ in the CV- group (70.4% vs 20%, *p*=0.009).

**Table 2:** - Clinical changes of subjects prior to and 3 months after EBV implantation.

Outcome	Baseline	3 months	Change from baseline to 3 months	*P*-value
Post-BD FEV1 (L)	0.85 ± 0.28	0.97 ± 0.28	0.12 ± 0.20	<0.001^†^
Post-BD FEV1 (% predicted)	30.78 ± 10.22	35.36 ± 11.06	4.59 ± 7.87	0.001^†^
Post-BD FVC (L)	2.27 ± 0.76	2.54 ± 0.72	0.27 ± 0.61	0.009^†^
Post-BD FVC (% predicted)	63.53 ± 20.28	72.29 ± 20.13	8.76 ± 17.86	0.005^†^
Post-BD FEV1/FVC (%)	38.17 ± 9.30	39.06 ± 9.27	0.89 ± 4.37	0.231
Post-BD RV (L)	6.12 ± 1.43	5.56 ± 1.65	-0.56 ± 1.05	0.008^†^
Post-BD RV (% predicted)	263.78 ± 62.75	236.71 ± 62.65	-27.07 ± 49.58	0.007^†^
Post-BD TLC (L)	8.75 ± 1.54	8.47 ± 1.73	-0.29 ± 0.92	0.107
Post-BD TLC (% predicted)	138.99 ± 23.81	134.88 ± 23.95	-4.11 ± 17.12	0.207
Post-BD RV/TLC (%)	69.76 ± 9.21	64.68 ± 9.13	-5.08 ± 11.25	0.022*
6MWD (m)	287.06 ± 98.54	351.92 ± 99.22	64.86 ± 60.45	<0.001^†^
mMRC (points)	3.0 [2.0, 3.0]	2.0 [2.0, 2.8]	-0.5 [-1.0, 0.0]	0.007^†^
Target lobe volume (ml)	1693 ± 790	1127 ± 676	-566 ± 741	<0.001^†^
Percentage of Subjects with Post-BD FEV1 (L) improvement of ≥15%	-	55.3% (21/38)	-	-
Percentage of Subjects with 6MWD improvement of ≥25 m	-	65.8% (25/38)	-	-
Percentage of subjects with TLVR ≥563ml	-	42.1% (16/38)	-	-
Percentage of subjects with RV reduction ≥430ml	-	57.9% (22/38)	-	-

Values are presented as mean ± standard deviation or median (interquartile range). post-BD: post-bronchodilator, FEV1: forced expiratory volume in 1 second, FVC: forced vital capacity, RV: residual volume;, TLC: total lung capacity, 6MWD: 6-minute walk distance, mMRC: modified Medical Research Council, ^*^ *p*<0.05, ^†^ *p*<0.01

### Chartis versus HRCT for clinical outcome prediction

The diagnostic performance of the Chartis System, CT fissure analysis, and a combination strategy (CV- and FI ≥90%) was compared. The combination strategy had the highest area under the curve (AUC): combination strategy (AUC=0.756, confidence interval (CI)=[0.592, 0.920], *p*=0.008); Chartis System (AUC=0.702, CI=[0.523, 0.881], *p*=0.037; CT fissure analysis (AUC=0.655, CI = [0.471, 0.838], *p*=0.111). The AUC values of the combination strategy and the Chartis system were greater than that of the CT fissure analysis (*p*<0.05). However, the difference between the combination strategy and the Chartis System was not statistically significant (*p*>0.05).

### Adverse events

During the 3-month follow-up period, there were no deaths. Table 3 summarized all adverse events occurred. The most common adverse events were coughing, increase in sputum production, and chest discomfort. Most of these presentations were mild or moderate and could be relieved by symptomatic treatment. Pneumothorax did not occur in any of the subjects. Seven serious adverse events occurred in 6 (15.8%) subjects. Three (7.9%) experienced a COPD exacerbation during the treatment period. All of these patients achieved remission after hospitalization. One patient coughed up one valve 2 months after EBV treatment and received a valve reimplantation. Pneumonia in a non-treated lobe occurred in one patient, and hiatus hernia occurred in another patient. Both of them were relieved after medication.

**Table 3:** - Adverse events occurred during 3 months after endobronchial valve treatment.

Adverse events	From the day of the procedure to 45 days afterward	Between the 45 days after the procedure and the 3-month follow-up date
***Respiratory***		
Cough	12 (31.6)	8 (21.1)
Increase in sputum production	10 (26.3)	6 (15.8)
Chest discomfort	7 (18.4)	4 (10.5)
Chest pain	1 (2.6)	0 (0.0)
Upper respiratory tract infection	1 (2.6)	0 (0.0)
Fever	0 (0.0)	1 (2.6)
Pneumonia	0 (0.0)	1 (2.6)
***Non-Respiratory***		
Acute urinary retention	2 (5.3)	0 (0.0)
Atrial fibrillation	1 (2.6)	0 (0.0)
Hiatus hernia	0 (0.0)	1 (2.6)

Eight patients received one revision bronchoscopy at the 3-month follow-up visit because of coughing or increased sputum production. Granulation tissue formation was found in 5 patients, 4 of which (80%) did not meet or exceed the MCID in FEV_1_. Valve replacement was performed in 2 patients for valve migration. One patient had the valves permanently removed due to personal request.

## Discussion

Endobronchial valves therapy has become one of the treatment options for patients with advanced emphysema. Our study showed that EBV therapy provided clinically meaningful improvements in exercise tolerance, lung function, and dyspnea in Chinese patients with advanced emphysema and no CV, with a low incidence of pneumothorax.

A statistically significant improvement in FEV_1_ was observed in patients with no CV. The absence of CV was generally considered as the key point for treatment success of EBV. However, in 2016, Herzog et al^5^ described the 4 Chartis phenotypes that were found in COPD patients: CV positive (CV+); CV-; LF and low plateau. Significant improvements in both FEV_1_ and target lung volume reduction were seen in both CV- and LF patients with an ipsilateral CV- lobe. Theoretically, the LF phenotype is considered to be attributed to a phenomenon called the “dynamic collapse”, and the effects of EBV on this type of patient remain unclear.^6,7^ In our study, only 20% of the subjects with LF achieved the MCID for FEV_1_, which means that the LF patient may not be considered as a potential candidate for EBV therapy.

In this study, the diagnostic performance of the Chartis system was superior to that of the CT fissure analysis. The combination strategy only caused a slight improvement in the predictor accuracy, however, some patients did lose some benefit from this method. Direct measurement of CV by the Chartis System was preferred for patient selection. Meanwhile, CV was an independent predictor for clinical benefit. Gompelmann et al^8^ reported similar accuracy for predicting TLVR using EBV between the Chartis system and HRCT (74% vs 77%). de Oliveira et al^9^ and Koster et al^10^ suggested 2 new combination criteria for patient selection. The latest expert panel recommendation is as follows: For patients with FI >95%, Chartis measurement is optional; for patients with 80-95% completeness of the fissure, Chartis measurement is recommended; for patients with FI <80%, EBV therapy is not suitable and Chartis evaluation is not needed.^11^ However, according to a recent study, Chartis assessment should always be taken in the right lung, even with a complete fissure (>95%).^12^ In general, more studies on the evaluation of the Chartis system and HRCT are needed for achieving a greater clinical benefit in a larger patient population.

Notably, pneumothorax did not occur post-procedure, which was different from that reported in recent randomized clinical trials showing a pneumothorax rate of 29.2-34.4%. The average percentage of emphysematous destruction in the ipsilateral lobe was only 27.7%, which was significantly lower than that found in previous studies. The incidence of pneumothorax might be increased by a worse ipsilateral lobe. Meanwhile, the smaller average TLVR in the LF group may have led to a total lower incidence of pneumothorax. It also might depend on the heterogeneity of lungs. In our study, all subjects had the heterogeneous emphysema (heterogeneity: 25.7% [15.3%, 34.6%]). Unfortunately, we did not find the correlation between degree of heterogeneity and the incidence of pneumothorax. It might be limited by the small sample size of the study cohort. Our results may be clinically meaningful, as pneumothorax is the most important post-procedural complication. A better ipsilateral lobe may be an effective way to reduce the rate of pneumothorax, which also needs to be confirmed in further research.

### Study limitation

First, this was a retrospective post-hoc analysis, even though EBV therapy was performed in accordance with the predetermined standardized strategies. Second, there was no assessment of quality of life due to the lack of data from the St. George’s Respiratory Questionnaire. Third, the follow-up period was only 3 months. Finally, the small sample size of the study cohort inevitably limited the statistical significance of the results. To confirm this study’s findings, additional prospective studies with a longer follow-up period are required.

In conclusion, patients with advanced emphysema and no collateral ventilation could benefit significantly from endobronchial valve therapy with a low incidence of pneumothorax. More studies are needed to further increase the proportion of patients who could achieve significant clinical benefits from EBV treatment.
